# A Multi-Engine Consensus Docking Pipeline for RNA Aptamer Screening Against ACC Oxidase (ACO): Statistical Validation, Machine Learning Analysis, and Pilot Cross-Target Evaluation Against ACC Synthase (ACS)

**DOI:** 10.3390/ijms27135947

**Published:** 2026-07-02

**Authors:** Héctor Ramón Martínez-de la Hoya, Cristian Patricia Cabrales-Arellano, Josué Ortiz-Medina, Efren Delgado, Juan Antonio Rojas-Contreras, Norma A. García-Vidaña, Damián Reyes-Jáquez, Rubén Guerrero-Rivera

**Affiliations:** 1 Division of Research and Postgraduate Studies, Tecnológico Nacional de México/I.T. Durango, Blvd. Felipe Pescador 1830, Durango 34080, Mexico; 15041230@itdurango.edu.mx (H.R.M.-d.l.H.); j.ortiz@itdurango.edu.mx (J.O.-M.); jrojas@itdurango.edu.mx (J.A.R.-C.); norma.garcia@itdurango.edu.mx (N.A.G.-V.); 2 Department of Biology, Eastern New Mexico University, Portales, NM 88130, USA; cristian.cabralesarellano@enmu.edu; 3 Department of Electrical and Electronics Engineering, Tecnológico Nacional de México/I.T. Durango, Blvd. Felipe Pescador 1830, Durango 34080, Mexico; 4 Department of Family and Consumer Sciences, New Mexico State University, Las Cruces, NM 88003, USA; edelgad@nmsu.edu; 5 Department of Chemical and Biochemical Engineering, Tecnológico Nacional de México/I.T. Durango, Blvd. Felipe Pescador 1830, Durango 34080, Mexico

**Keywords:** RNA aptamers, ACC oxidase, ACC synthase, ethylene biosynthesis, consensus docking, HADDOCK3, machine learning, AptaDock

## Abstract

Postharvest losses in climacteric fruits are largely driven by ethylene, and inhibiting its biosynthesis is an active research goal. RNA aptamers are attractive candidates for modulating the two rate-limiting enzymes—ACC oxidase (ACO) and ACC synthase (ACS), without the toxicity concerns of chemical inhibitors. We built a computational pipeline using three independent docking engines: ITD DOCK (Graphics Processing Unit [GPU]-accelerated, Assisted Model Building with Energy Refinement [AMBER] force fields), HDOCK Lite (knowledge-based geometric potentials), and HADDOCK3 (semi-flexible refinement), screened against large aptamer libraries. Applied to ACC oxidase with 9813 aptamers, engine scores were largely complementary, with all pairwise correlations statistically non-significant on the full dataset (Spearman |ρ|≤0.013, p>0.20 for all pairs, n=9813). A Random Forest on sequence-only features failed to predict docking scores ($R^{2}=-0.012$, Root Mean Square Error [RMSE] = 21.79 kcal/mol), while a positive-control model on HADDOCK3 energy components achieved $R^{2}=0.991$, confirming that predictive information only becomes available after docking. A preliminary pilot screening against ACC synthase (n=97 valid complexes) suggested pipeline generalizability to a second structurally distinct target and identified two putative dual-active candidates, pending confirmation against the full library. The complete workflow is automated in AptaDock, a standalone desktop application.

## 1. Introduction

Every year, roughly 45% of fruit and vegetable production is lost before it reaches consumers. A large part of that loss in climacteric fruits (tomatoes, bananas, avocados) comes down to one molecule: ethylene ($C_{2}H_{4}$), a gaseous plant hormone that sets off the entire ripening and senescence cascade [1]. Cold storage, modified atmospheres, and chemical treatments like 1-methylcyclopropene (1-MCP) are the standard tools to slow this process, but they come with real drawbacks: high energy costs, regulatory concerns about residues, and limited accessibility for smaller producers [2,3].

Aptamers, short single-stranded oligonucleotides that fold into three-dimensional shapes capable of gripping specific protein targets, have attracted attention as a cleaner alternative [4,5,6,7]. They can reach nanomolar affinities, they are chemically stable, and they can be designed computationally rather than isolated through months of experimental SELEX rounds [8,9,10,11]. Applications against viral proteins and cancer targets have shown that in silico design actually works in practice [12,13,14].

A related enzyme worth noting is the Ethylene Forming Enzyme (EFE), a non-heme Fe(II) and 2-oxoglutarate-dependent oxygenase found in certain bacteria that produces ethylene through a mechanistically distinct route [15,16,17]. While EFE and ACO share ethylene as a product, they differ substantially in cofactor requirements, substrate specificity, and active site architecture, making them independent targets for inhibition strategies.

A recent paper from our group (Aparicio-Breceda et al.) explored exactly this idea, using ZDOCK and HDOCK to dock aptamer candidates against ACC synthase and ACC oxidase [18]. That work established that rigid-body docking is a feasible starting point for this problem. What it did not include was semi-flexible refinement, any statistical test of whether the two docking servers were actually giving independent information, feature importance analysis, or a dataset large enough to train machine learning models. This paper picks up where that one left off.

The core idea here is simple: instead of trusting a single docking method, we run three engines that work from completely different physical principles and take consensus. If two methods are highly correlated, one of them is redundant, since the whole point of using multiple methods is that they are not. We also wanted to know whether it would be possible to skip docking entirely and predict docking scores from sequence features alone. It cannot be done, and documenting exactly why is one of the main contributions of this paper.

### 1.1. Ethylene Biosynthesis Pathway

Ethylene biosynthesis runs through the Yang cycle and depends on two enzymatic steps. ACC synthase (ACS) converts S-adenosyl-L-methionine (SAM) into 1-aminocyclopropane-1-carboxylic acid (ACC), and ACC oxidase (ACO) then converts ACC into ethylene. Both steps are rate-limiting, which makes both enzymes interesting targets. Crystallographic studies have resolved the active sites of ACS [19] and ACO [20] in enough detail for structure-based design. The reaction mechanism of ACO has been investigated computationally using density functional theory [21], and the enzyme has been characterized experimentally through EPR spectroscopy, mutational analysis, and bioconjugation studies [22,23]. Small molecule inhibitors have been found for both enzymes [24,25], which confirms that the binding sites are druggable, a useful sanity check before investing in an aptamer campaign.

### 1.2. Computational Aptamer Discovery

The basic idea in computational aptamer design is to generate large sequence libraries and dock them against the target, scoring each candidate to find the best binders [14,26,27]. The challenge is that binding is driven by three-dimensional structure, not sequence alone, so you need three-dimensional models of every aptamer before you can dock it. Tools like RNAComposer have made automated structure prediction practical at scale [28,29]. The remaining bottleneck is the docking itself: traditional stochastic search algorithms are too slow for libraries of thousands of candidates [30,31]. Recent developments have extended these pipelines with deep learning-based design tools and diffusion models for aptamer generation [32,33].

### 1.3. FFT-Based Protein-RNA Docking

One solution to the speed problem is to use Fast Fourier Transform (FFT) methods. The Katchalski-Katzir algorithm [34] discretizes both molecules onto grids and computes their interaction through convolution in the frequency domain, reducing the translational search from $O(N^{6})$ to $O(N^{3}\log N)$. ITD DOCK, the engine we developed for this work, implements this approach on GPU hardware through CUDA, adding AMBER-based local refinement for the top candidates from the global search.

### 1.4. Machine Learning for Docking Score Prediction

Our main goal was generating a high-quality, multi-dimensional docking dataset to train predictive models. Random Forest regression [35] is a practical first choice for this kind of analysis because it handles non-linear feature interactions and provides interpretable importance scores. We used it here to answer a specific question: can you predict docking scores before running any docking, from sequence features alone? It cannot, and understanding why took up a significant part of this work.

## 2. Results and Discussion

### 2.1. Are the Three Engines Actually Independent?

The value of using three engines depends entirely on whether they are measuring different things. We assessed this in two stages. First, we computed pairwise correlations on a representative pilot set of 100 aptamers randomly sampled from the full ACO library (Table 1). Second, to confirm that the pilot results were representative and to address the reviewer concern about small-sample reliability, we repeated the full correlation analysis on the complete filtered ACO dataset (n=9813; Table 2).

#### 2.1.1. Correlation Analysis for ACC Oxidase

Table 1 shows the results. The pilot analysis (Table 1) shows that most correlations are non-significant. The only borderline result was HDOCK Lite–HADDOCK3 (Kendall τ=−0.139, p=0.040, n=100). However, the full-dataset analysis (Table 2, n=9813) shows that all three pairwise correlations are indistinguishable from zero (all |r|<0.015, all p>0.20). The borderline pilot result did not replicate, confirming it was a small-sample artefact. The engines are, therefore, largely complementary across the entire screened library. Across all three pairs, no correlation reaches statistical significance (all p>0.20, all |r|<0.014), providing strong evidence that the three engines capture complementary dimensions of the binding landscape. The pilot analysis on 100 randomly sampled aptamers was consistent with these full-dataset results, confirming that the random sample was representative of the library.

**Figure 1:**
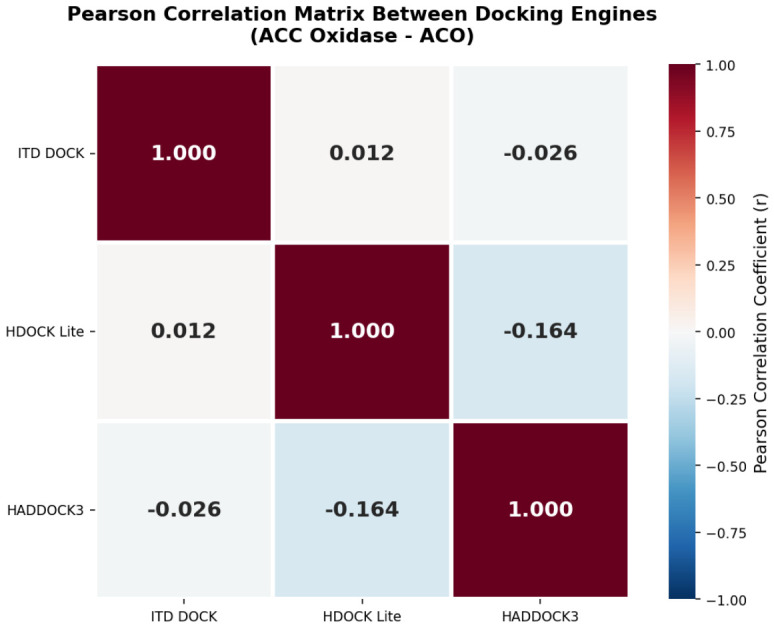
Pearson correlation matrix between the three docking engines for ACC oxidase. All values are close to zero and statistically non-significant (n=9813): ITD DOCK vs. HDOCK Lite r=−0.007; ITD DOCK vs. HADDOCK3 r=0.009; HDOCK Lite vs. HADDOCK3 r=−0.008. This confirms that the three scoring functions are capturing complementary aspects of binding.

**Figure 2:**
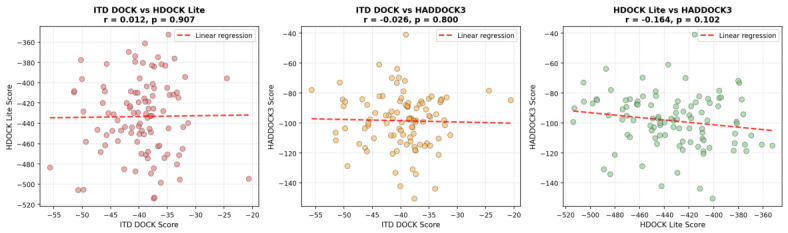
Pairwise scatter plots for ACC oxidase with linear regression lines (dashed red). **Left**: ITD DOCK vs. HDOCK Lite; **Center**: ITD DOCK vs. HADDOCK3; **Right**: HDOCK Lite vs. HADDOCK3. No meaningful linear trends are visible in any pair, consistent with the low correlations.

**Figure 3:**
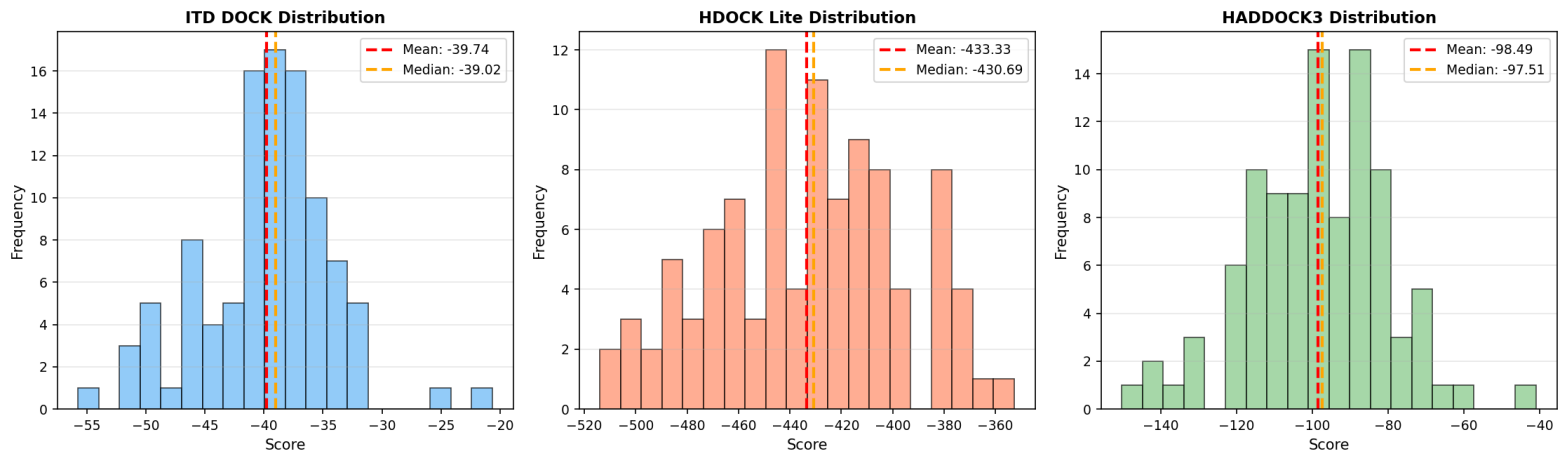
Score distributions for 100 representative ACC oxidase aptamers. **Left**: ITD DOCK (mean −39.74 kcal/mol, median −39.02 kcal/mol); **Center**: HDOCK Lite (mean −433.33 kcal/mol, median −430.69 kcal/mol); **Right**: HADDOCK3 (mean −98.49 kcal/mol, median −97.51 kcal/mol). The three engines operate on entirely different numerical scales, which is another reason averaging raw scores directly would be meaningless.

**Figure 4:**
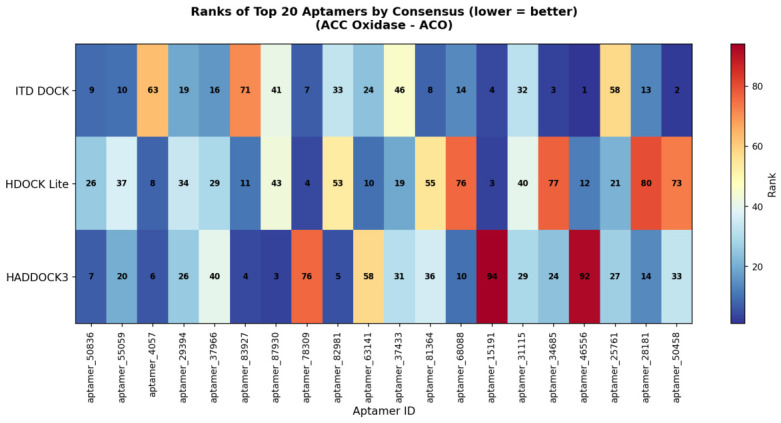
Consensus ranking heatmap for the top 20 ACO aptamers (n=100). Each column is one aptamer; rows show its rank within each engine (1 = best, 100 = worst). The mixed color pattern, where no aptamer dominates all three engines simultaneously, illustrates why rank-based consensus rather than any single engine is needed to identify robust candidates.

Figure 1, Figure 2, Figure 3 and Figure 4 show the correlation heatmap, pairwise scatter plots, score distributions, and consensus ranking heatmap for ACC oxidase.

#### 2.1.2. What Low Correlations Actually Mean

Low correlations between engines are not a problem with the pipeline; they are exactly what the pipeline was designed to achieve. If ITD DOCK and HDOCK Lite scored aptamers identically, we would be running two versions of the same calculation. The observed independence means each engine is capturing something the others miss: ITD DOCK evaluates explicit AMBER electrostatics; HDOCK Lite recognizes geometries that are statistically common in the PDB; HADDOCK3 accounts for how the binding site deforms to accommodate the aptamer. An aptamer that slips through one engine’s filter but gets caught by the other two is unlikely to make the final shortlist, which is the point of taking consensus.

### 2.2. Feature Importance Analysis (ACC Oxidase, n = 9813)

#### 2.2.1. Analysis A: Can We Skip Docking Entirely?

The obvious question is whether a simpler model could replace the pipeline. If docking scores could be predicted from sequence features alone, there would be no need to run hours of docking per aptamer. We trained a Random Forest on 48 features derived purely from sequence and secondary structure—nucleotide and dinucleotide frequencies, GC content, thermodynamic folding energy, net charge, structural descriptors—with HADDOCK3 energy components explicitly excluded.

The model achieved $R^{2}=-0.012$ (RMSE = 21.79 kcal/mol) on the held-out test set. A negative $R^{2}$ means the model does worse than simply predicting the dataset mean for every aptamer. It failed. The most informative single feature was ΔG (importance 6.9%), but it had a univariate correlation of only r=0.012 (p=0.23) with HADDOCK score, essentially zero. This makes sense: whether an aptamer folds stably and whether it binds well to a specific protein are largely unrelated questions, because stable folding does not guarantee that the folded shape fits the binding site.

#### 2.2.2. Analysis B: Positive Control

As a positive control, we trained a second model using only the four HADDOCK3 energy outputs (VdW, Elec, Desolv, BSA). Three of these terms (VdW, Elec, Desolv) appear directly in the HADDOCK score formula; BSA is not a formula component but correlates strongly with the composite score because larger buried interfaces drive more favorable interaction energies. This model is, therefore, expected to perform well by construction; it is recovering a known mathematical structure, not discovering anything new. Its value is purely contrastive: to show that Random Forest works correctly when a real signal is present, and that the failure of Analysis A, therefore, reflects a genuine absence of predictive information in sequence features, not a failure of the algorithm.

**Figure 5:**
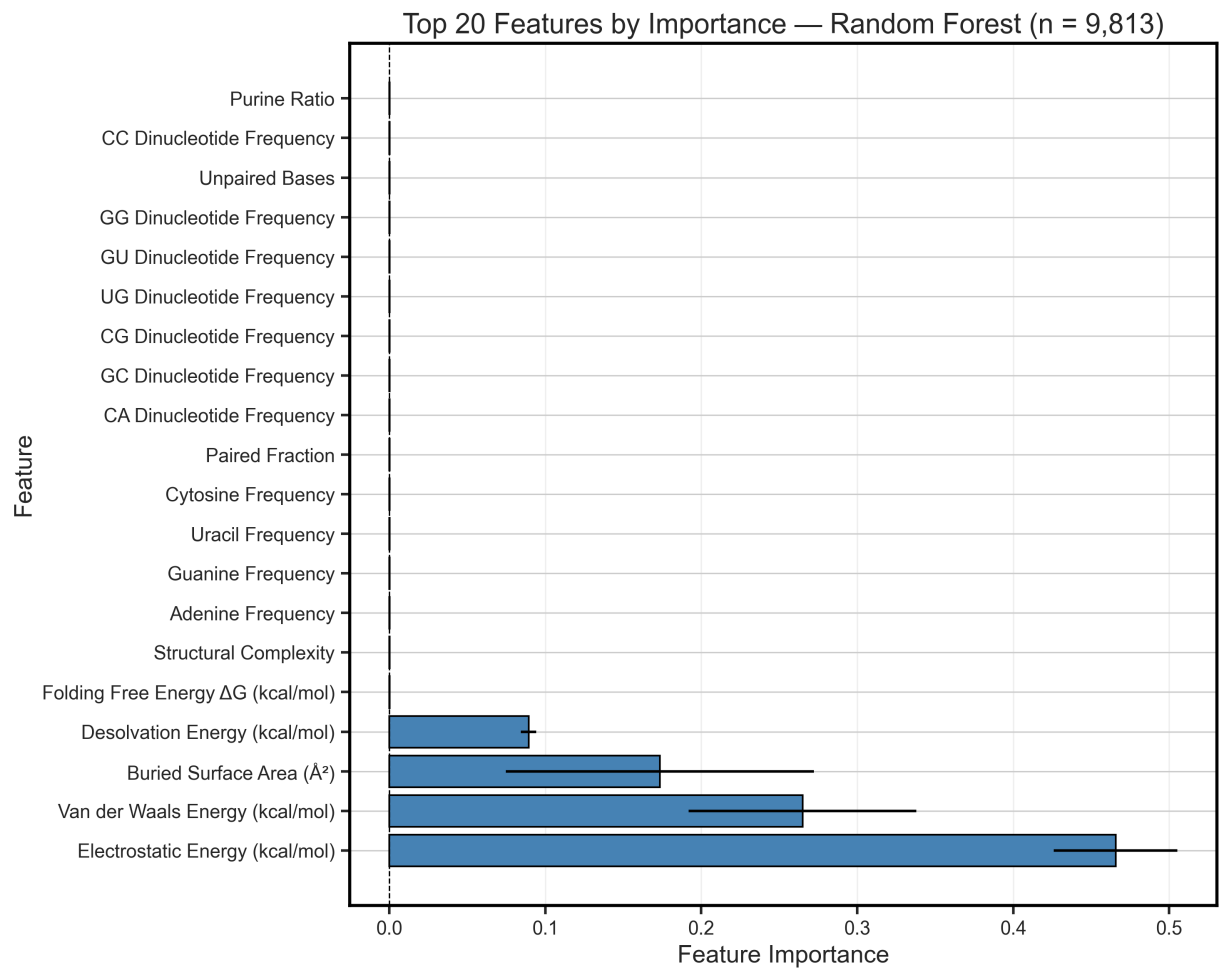
Feature importance from Analysis B (n=9813 aptamers). The four HADDOCK3 energy terms (Elec: 46.3%, VdW: 26.8%, BSA: 17.5%, Desolv: 9.4%) account for essentially all feature importance (>99%). The sequence-only baseline (Analysis A, 48 features, no energy terms) achieves $R^{2}=-0.012$, performing below the trivial mean predictor.

It achieved $R_{test}^{2}=0.991$ (RMSE = 2.06 kcal/mol). Feature importance rankings (Table 3, Figure 5) show electrostatics as the dominant term (Elec: 46.3%), followed by VdW (26.8%), buried surface area (BSA: 17.5%), and desolvation (Desolv: 9.4%).

The contrast between Analysis A ($R^{2}=-0.012$) and Analysis B ($R^{2}=0.991$) is the main result. Analysis A shows that sequence-derived features carry no exploitable information for predicting docking scores; Analysis B confirms the algorithm is working correctly by recovering the known mathematical structure of the HADDOCK score from its own components. Together, they show that the information needed to reproduce a docking score only becomes available after the docking is actually performed. This is why the tripartite pipeline cannot be shortcut with sequence-based pre-screening at the current state of the field.

### 2.3. Univariate Correlation Analysis (ACC Oxidase, n = 9813)

To complement the Random Forest results, we looked at how each feature correlates individually with the HADDOCK score using Pearson correlation coefficients.

#### 2.3.1. Energy Components Correlate Strongly

**Figure 6:**
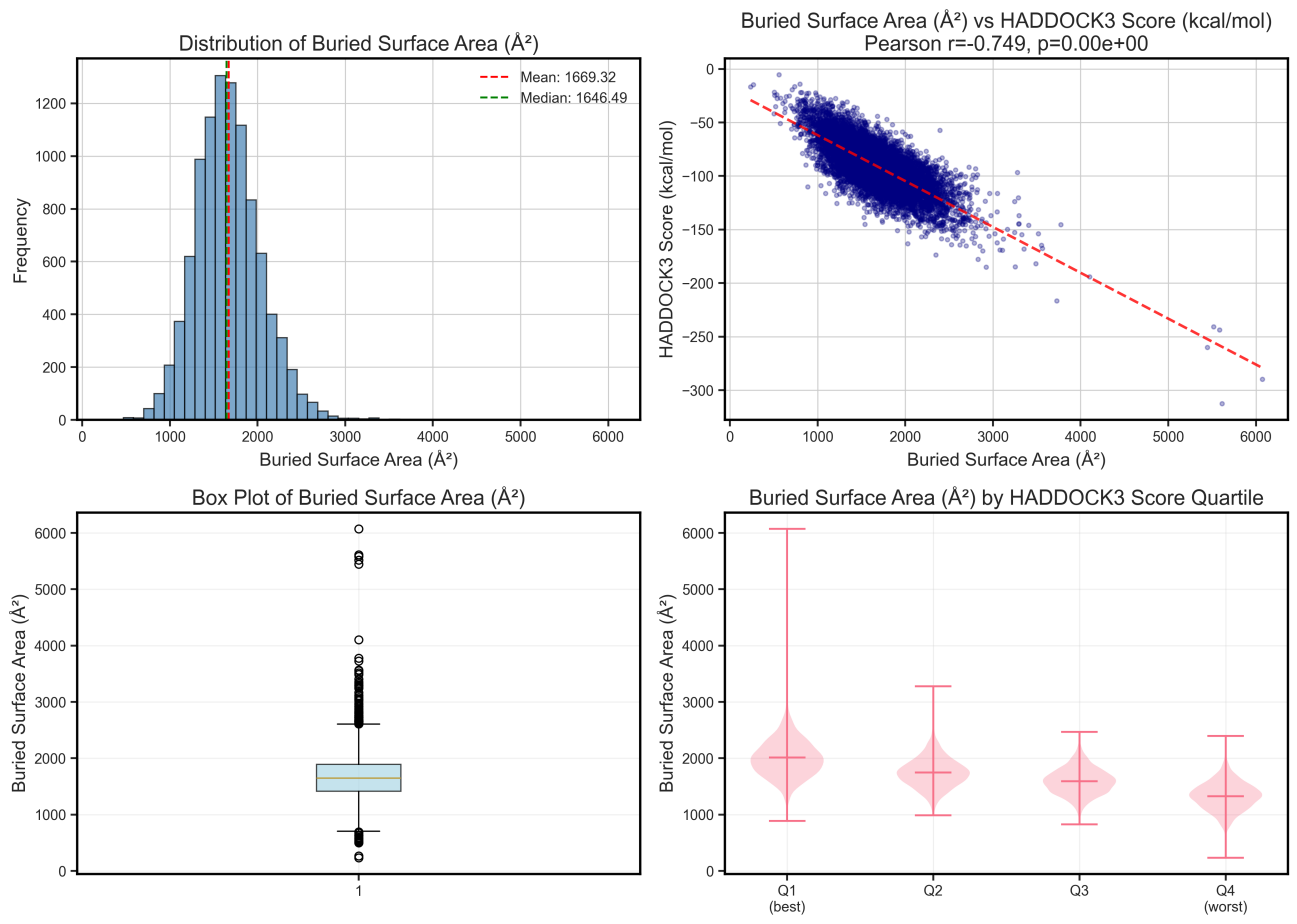
BSA versus HADDOCK score (n=9813). Scatter plot shows a strong negative correlation (r=−0.749, $p<10^{-300}$). Violin plots by score quartile confirm that top-scoring aptamers (Q1, median ≈ 1982 Å^2^) make systematically larger contact interfaces than poor binders (Q4, median ≈ 1331 Å^2^).

**Figure 7:**
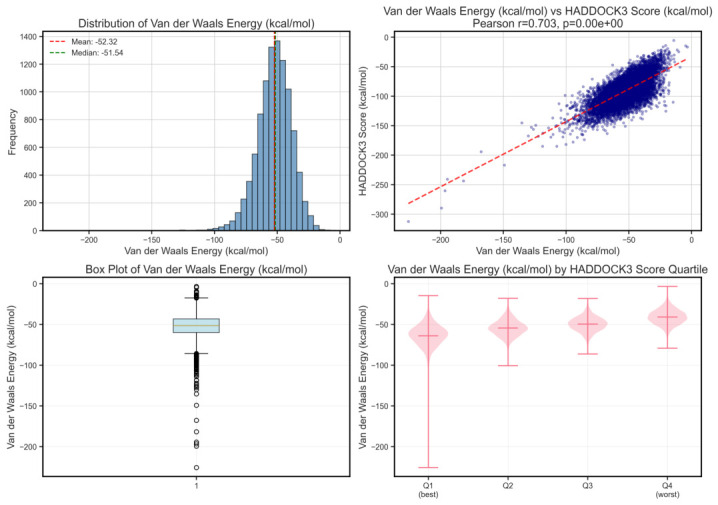
VdW energy versus HADDOCK score (n=9813). Correlation r=0.703 ($p<10^{-300}$). Q1 aptamers have more favorable van der Waals contacts (median ≈−63 kcal/mol) than Q4 aptamers (median ≈−41 kcal/mol).

**Figure 8:**
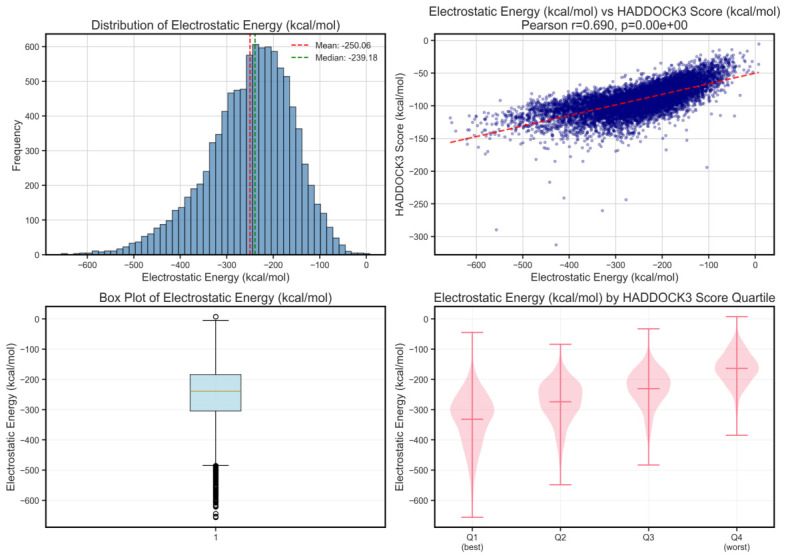
Electrostatic energy versus HADDOCK score (n=9813). Correlation r=0.690 ($p<10^{-300}$). The broad distribution reflects a wide range of charge complementarity patterns across the library. Q1 aptamers show substantially stronger electrostatic interactions (median −319 kcal/mol) than Q4 (median −162 kcal/mol).

**Figure 9:**
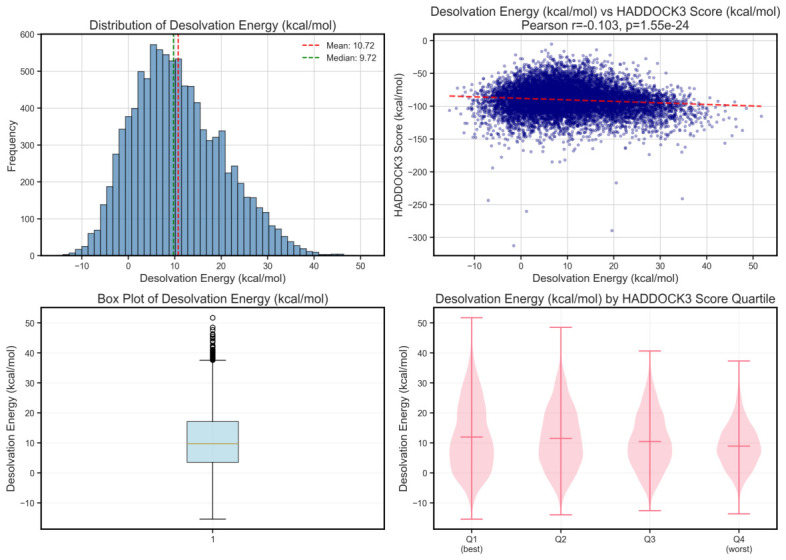
Desolvation energy versus HADDOCK score (n=9813). The correlation is weak (r=−0.103, $p=1.55\times 10^{-24}$) but statistically significant due to the large sample size. The effect is considerably smaller than BSA, VdW, or Elec.

Three of the four HADDOCK3 energy components show strong individual correlations with the final score (Figure 6, Figure 7, Figure 8 and Figure 9):

Buried Surface Area (BSA) has the strongest correlation at r=−0.749 ($p<10^{-300}$). The negative sign means that aptamers making larger contact interfaces with the protein also get more favorable (more negative) HADDOCK scores, which is physically intuitive: a bigger, better-fitting contact surface drives binding. Aptamers in the top scoring quartile (Q1) have a median BSA of approximately 1982 Å^2^, compared to about 1331 Å^2^ for the worst quartile (Q4).

Van der Waals energy (VdW) correlates at r=0.703 ($p<10^{-300}$). The positive sign reflects HADDOCK3’s sign convention: more negative VdW energies correspond to stronger attractive interactions, which in turn produce more negative (better) scores. The Q1 median VdW is approximately −63 kcal/mol versus −41 kcal/mol for Q4.

Electrostatics (Elec) follows a similar pattern, r=0.690 ($p<10^{-300}$), with Q1 aptamers showing much stronger charge complementarity (median −319 kcal/mol) than Q4 (median −162 kcal/mol).

Desolvation (Desolv) is significant but weak, r=−0.103 ($p=1.55\times 10^{-24}$). It matters, but it is not the main driver.

#### 2.3.2. Sequence and Thermodynamic Features Show No Correlation

**Figure 10:**
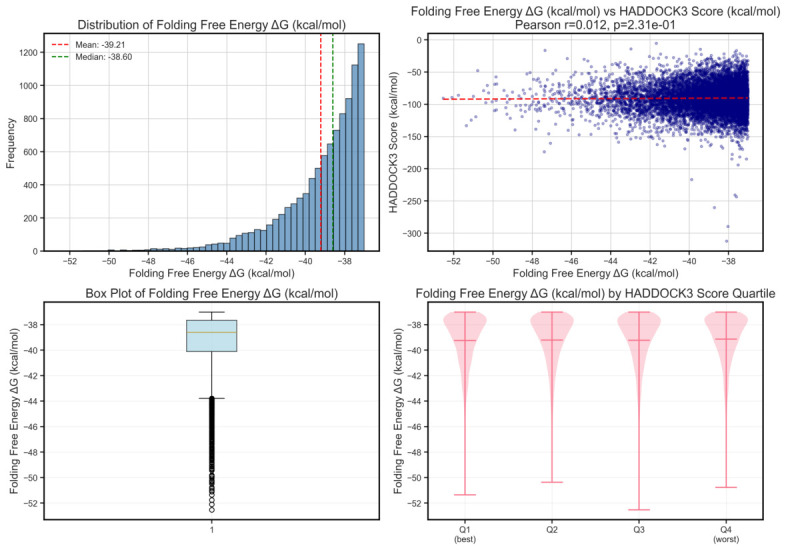
Folding free energy (ΔG) versus HADDOCK score (n=9813). Near-zero correlation (r=0.012, p=0.23, not significant). The violin plots confirm similar ΔG distributions across all score quartiles, supporting the use of ΔG as a structural stability filter but not as a binding predictor.

In contrast to the energy components, sequence-derived features are essentially flat (Figure 10). Folding free energy ΔG has a correlation of r=0.012 (p=0.23, not significant) with HADDOCK score. Looking at the violin plots, aptamers in all four scoring quartiles have nearly identical ΔG distributions. A stable aptamer does not bind better; stability and shape complementarity are independent properties.

This is consistent with the Analysis A Random Forest result ($R^{2}=-0.012$): the near-zero individual correlations are not a linearity artifact, they reflect a genuine absence of predictive information in sequence features.

### 2.4. Principal Component Analysis and Clustering (ACC Oxidase, n = 9813)

#### 2.4.1. The Dataset Is Genuinely High-Dimensional

**Figure 11:**
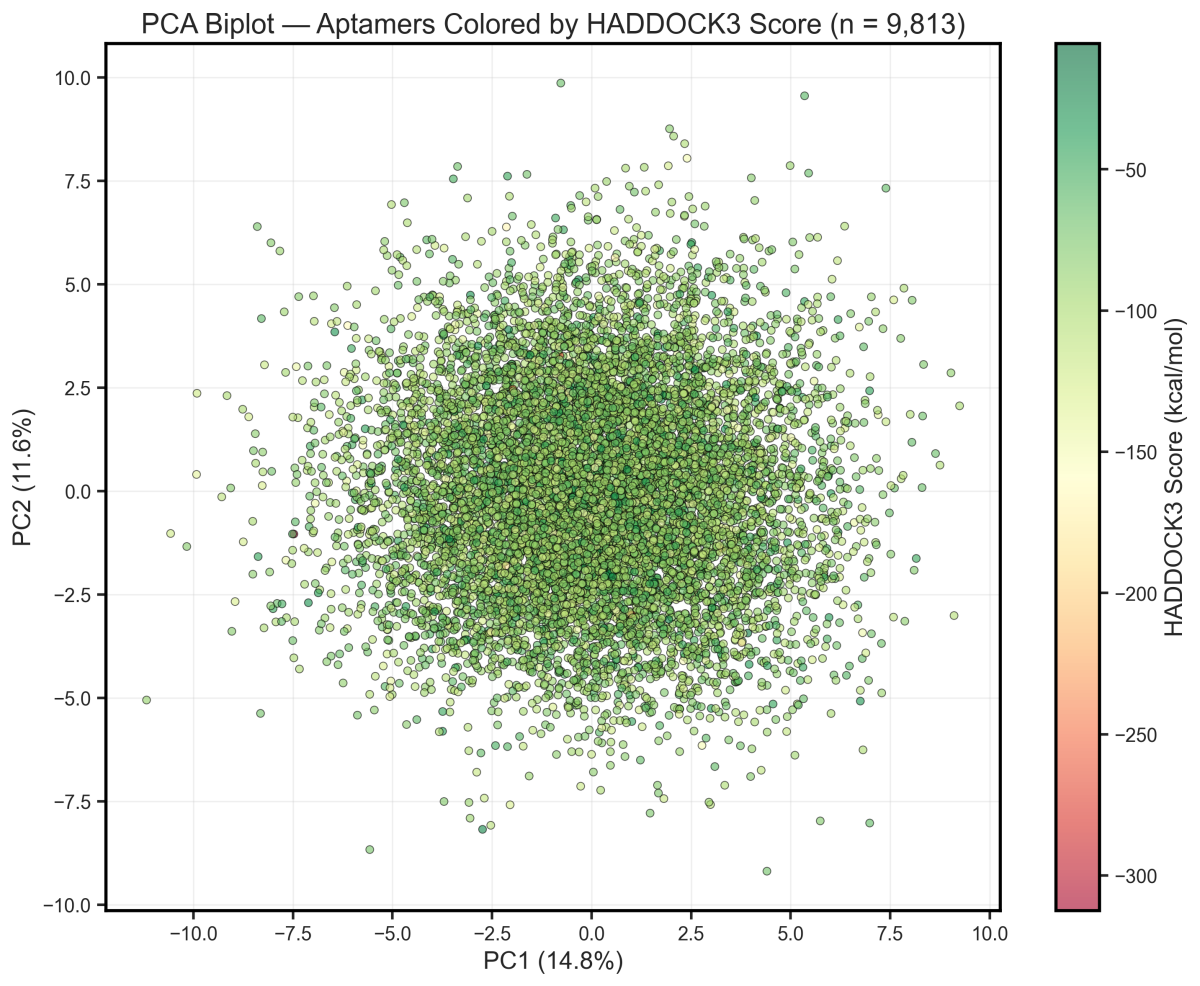
PCA biplot of 9813 aptamers colored by HADDOCK score. PC1 (14.8%) and PC2 (11.6%) explain 26.4% of total variance. The wide scatter reflects the multi-dimensional nature of binding affinity.

PCA on the full feature set reveals that just two principal components explain only 26.4% of the variance (PC1: 14.8%, PC2: 11.6%). You need 10 components to reach 65.7%. This is not a failure of the analysis; it confirms that binding affinity is a multi-dimensional phenomenon and that no two-dimensional projection captures it adequately. Figure 11 shows the aptamers projected onto PC1-PC2, colored by HADDOCK score. A subtle gradient is visible, but the scatter is large, which is consistent with the predictability results: there is signal, but it requires multiple dimensions to resolve.

#### 2.4.2. Five Distinct Binding Profiles

**Figure 12:**
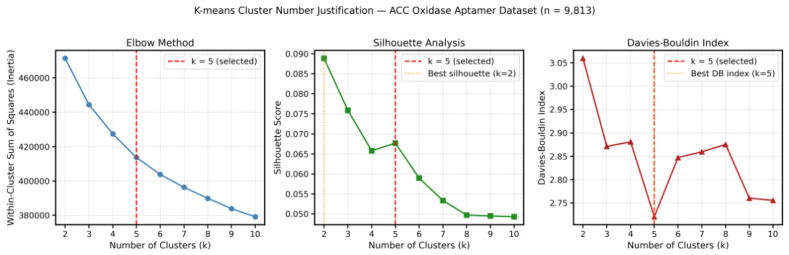
Cluster number selection for K-means analysis (n=9813 aptamers). Three complementary metrics were evaluated across k=2 to k=10. **Left**: elbow analysis (within-cluster sum of squares). **Center**: silhouette score. **Right**: Davies–Bouldin index. k=5 achieves the global minimum of the Davies–Bouldin index (DB=2.720, compared to 2.871 for k=3 and 2.847 for k=6) and a local maximum in the silhouette score (s=0.068, compared to 0.066 for k=4 and 0.059 for k=6), indicating that k=5 provides the optimal balance between intra-cluster cohesion and inter-cluster separation at a biologically interpretable granularity.

**Figure 13:**
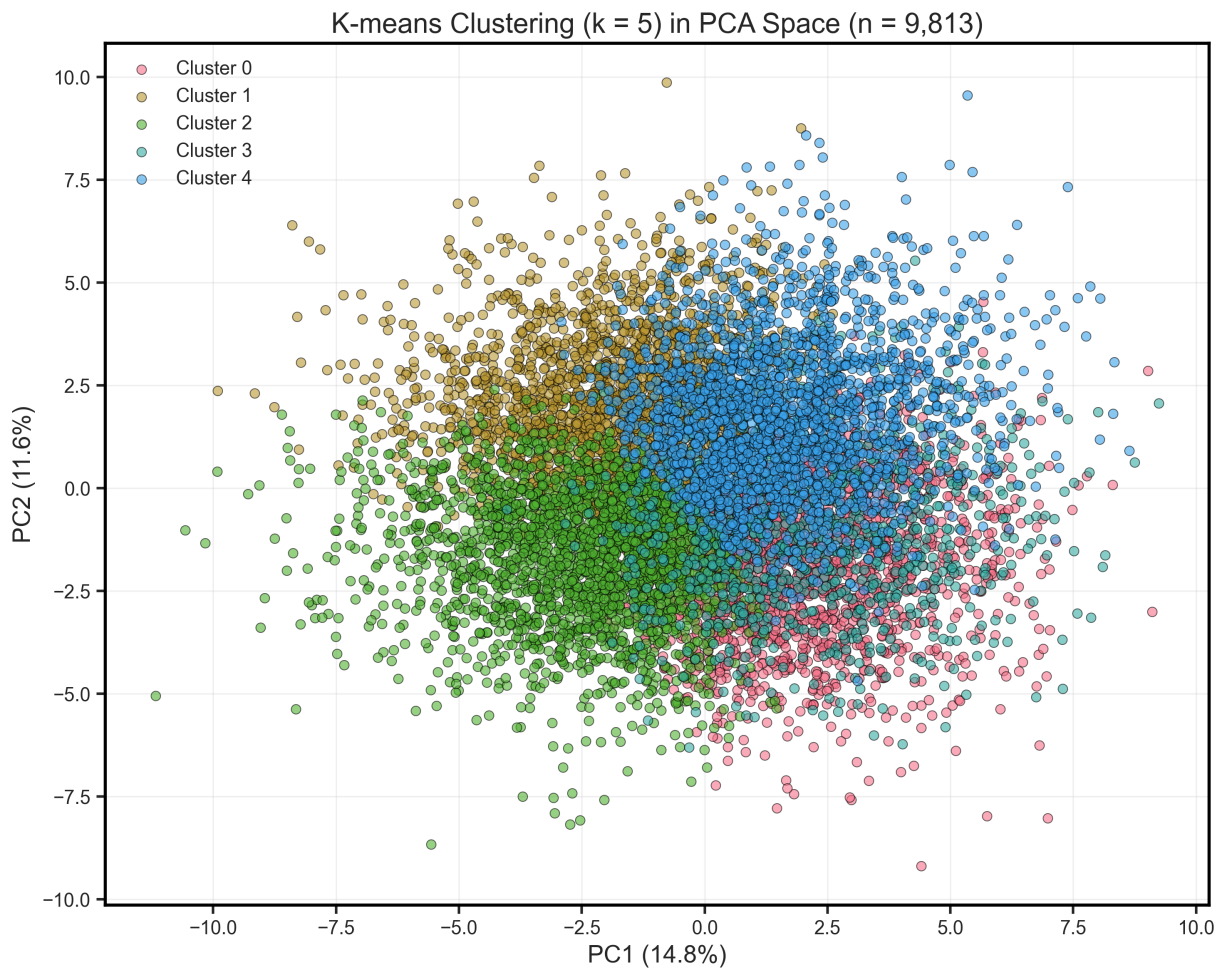
K-means clustering (k=5) of 9813 aptamers in PCA space. The five groups show statistically distinct mean HADDOCK scores, BSA values, and energy contribution profiles, suggesting multiple viable binding strategies.

K-means clustering (k=5, justified by Davies–Bouldin and silhouette analysis; see Figure 12) applied to the clean dataset (n=9813) identifies five aptamer groups with statistically distinct binding characteristics (Figure 13). The groups differ primarily in their combination of electrostatic and van der Waals contributions: one cluster is dominated by strong electrostatics and large BSA (mean HADDOCK score around −102 kcal/mol), two clusters show moderate balanced binding profiles (means around −95 to −98 kcal/mol), one cluster has lower electrostatic contributions (−93 kcal/mol), and one shows higher desolvation penalties (−96 kcal/mol).

The existence of multiple groups with different energy profiles is useful because it suggests that different docking engines may be more sensitive to different binding modes, which is another reason why using all three, rather than just one, improves the quality of the final ranking.

### 2.5. Design Rules from Decision Tree Analysis

We trained a decision tree (maximum depth = 5) on the full ACO dataset to extract interpretable thresholds that characterize high-affinity binders. Three representative rules from the tree illustrate the main patterns:

**Rule 1 (Excellent Affinity):** IF Elec ≤−221.36 kcal/mol AND BSA >1906.93 Å^2^ AND VdW ≤−86.63 kcal/mol ⇒ predicted HADDOCK score ≈−153 kcal/mol. All three energy terms need to be favorable simultaneously to reach this level.

**Rule 2 (Moderate Affinity):** IF −273.26< Elec ≤−221.36 kcal/mol AND BSA ≤1906.93 Å^2^ AND VdW >−47.62 kcal/mol ⇒ predicted score ≈−79.80 kcal/mol. Strong electrostatics alone, without a big enough interface or strong VdW, gets you to moderate binding but not exceptional.

**Rule 3 (Low Affinity):** IF Elec >−221.36 kcal/mol AND VdW >−38.43 kcal/mol ⇒ predicted score ≈−32.65 kcal/mol. Weak interactions across the board lead to poor binding.

The consistent message is that no single feature is sufficient for high affinity. The best-scoring aptamers in this dataset combine strong electrostatics, a large contact surface, and favorable van der Waals contacts all at once. These threshold values are empirical rules derived specifically from the HADDOCK3 scoring function applied to this aptamer library and these enzyme targets; they reflect the mathematical structure of the HADDOCK score and should not be interpreted as universal design rules for RNA–protein binding. Their practical utility is in providing a data-driven starting point for prioritizing candidates for experimental testing within the framework of this pipeline.

### 2.6. Can a Deep Learning Model Do What the Random Forest Could Not?

We trained a multi-task Transformer on the same sequence and secondary structure features used in Analysis A, asking it to predict both the HADDOCK score and the HDOCK score simultaneously. Table 4 shows what happened.

It also failed, and in the same way. The model converged to predicting a nearly constant value close to the dataset mean regardless of input—a behavior known as collapse to the mean, which happens when the input features contain no meaningful signal about the target variable. Predictions varied over a range of only about 8 kcal/mol while the actual scores span 267 kcal/mol.

The 6.3% relative error (MAE/range) looks small but is misleading: any model that predicts the mean for every sample achieves similar relative error when the score distribution is bell-shaped. The appropriate metrics here are $R^{2}$ and Pearson *r*, and both indicate near-zero predictive capacity.

The fact that a Transformer with RoPE, SwiGLU, and RMSNorm components fails in exactly the same way as a classical Random Forest is informative. The problem is not the architecture; it is the input. Docking scores are determined by the three-dimensional geometry of the complex, and that information simply does not exist in the nucleotide sequence.

### 2.7. Validation on ACC Synthase

To check whether the pipeline holds up on a different target, we ran the same 100 aptamers through the full tripartite protocol against ACC synthase. Three complexes produced anomalous positive ITD DOCK scores (aptamer_80458: +311.9, aptamer_31115: +2225.8, aptamer_67633: +4013.0 kcal/mol), consistent with failures in the global FFT search rather than real binding predictions. These three were excluded, leaving n=97 valid complexes for all ACS analyses.

#### 2.7.1. Engine Independence Also Holds for ACS

Table 5 shows the results. The pattern is essentially the same as for ACC oxidase: most correlations are not significant. The only difference is that HDOCK Lite and HADDOCK3 show a weak but significant Spearman correlation (ρ=0.235, p=0.021) for ACS, which was not significant for ACO. This likely reflects differences in the active site geometry between the two enzymes rather than a problem with the pipeline. The three engines remain largely complementary.

**Figure 14:**
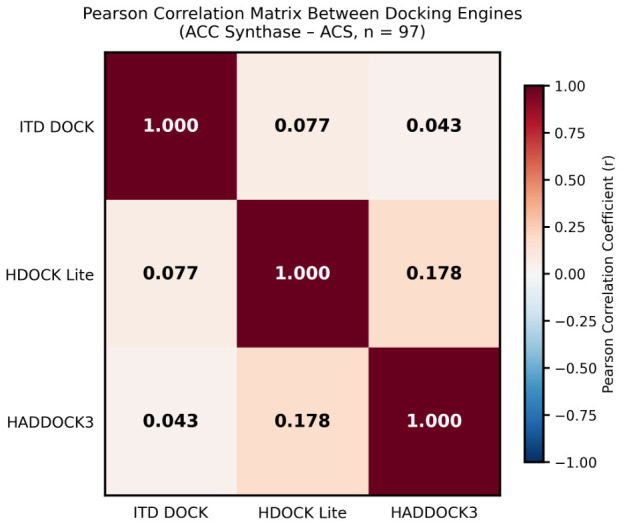
Pearson correlation matrix for ACC synthase (n=97, after excluding three ITD DOCK failures). The pattern mirrors the ACO result, confirming that the three engines remain largely complementary on a second target with a different active site geometry.

**Figure 15:**
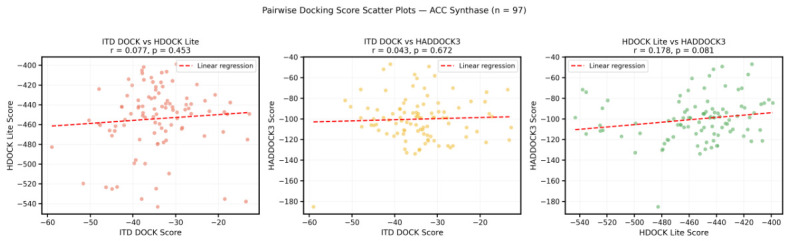
Pairwise scatter plots for ACC synthase (n=97). Weak, largely non-significant pairwise correlations across most engine pairs confirm the multi-engine approach is valid for this second target.

**Figure 16:**
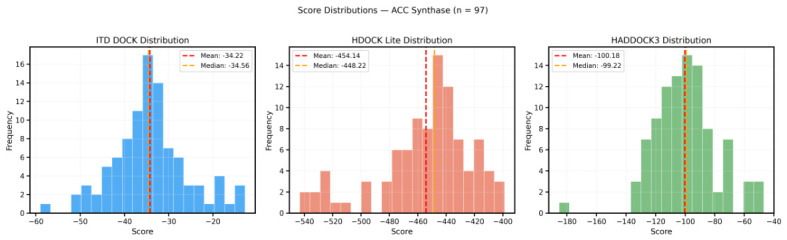
Score distributions for 97 valid ACC synthase complexes (three ITD DOCK failures excluded). **Left**: ITD DOCK (mean −34.22 kcal/mol, median −34.56 kcal/mol); **Center**: HDOCK Lite (mean −454.14, median −448.22); **Right**: HADDOCK3 (mean −100.18 kcal/mol, median −99.22 kcal/mol).

**Figure 17:**
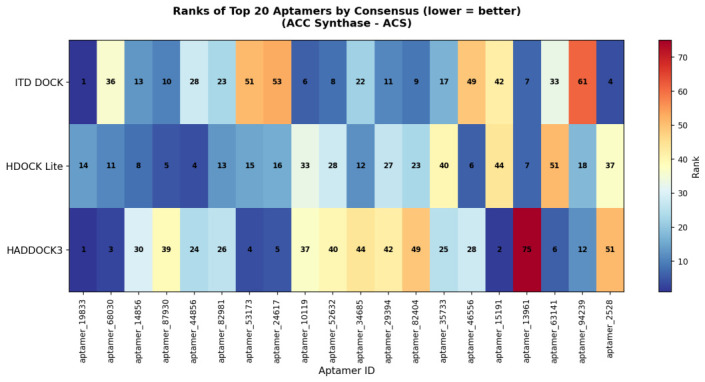
Consensus ranking heatmap for the top 20 ACS aptamers (n=97). The mixed color pattern, where no aptamer dominates all three engines simultaneously, mirrors the ACO result and confirms that the consensus is integrating genuinely different information from each engine.

Figure 14, Figure 15, Figure 16 and Figure 17 show the correlation matrix, scatter plots, score distributions, and consensus ranking heatmap for ACC synthase.

#### 2.7.2. Two Aptamers Rank Well Against Both Enzymes

Comparing the top 10 consensus candidates for ACO and ACS (Table 6), two aptamers (aptamer_87930 and aptamer_82981) appear in both lists. Given the limited size of the ACS pilot dataset (n=97), this overlap should be treated as a preliminary observation rather than a confirmed finding: the probability of two aptamers appearing in both top-ten lists by chance alone is not negligible in a sample of this size, and the result requires validation against the full ACS library before strong conclusions can be drawn. With that caveat, these two represent the highest-priority candidates for experimental follow-up if the goal is simultaneous inhibition of both rate-limiting steps of ethylene biosynthesis. The other eight top candidates per enzyme appear selective, which is also useful for applications where modulating only one step of the pathway is preferred.

Among the 20 top-ranked positions (top-ten per enzyme), 16 (80%) correspond to enzyme-selective aptamers: eight unique ACO-selective and eight unique ACS-selective. The two putative dual binders (aptamer_87930 and aptamer_82981) each appear in both top-ten lists, accounting for the remaining four positions (20%). This preliminary selectivity pattern is consistent with what has been reported for aptamers against closely related targets [36], and provides a testable hypothesis for future full-library cross-target screening.

### 2.8. Why Three Engines?

The most common objection to multi-engine docking is that it is computationally expensive and redundant if the methods agree with each other. The correlation analysis shows they do not agree, at least not for this system: pairwise Spearman correlations between the three engines are near zero for ACC oxidase and remain largely non-significant for ACC synthase. This is expected: the three engines are built on fundamentally different physical principles.

HDOCK Lite and HADDOCK3 form the validated core of the consensus: both have been benchmarked extensively by the community, and their scoring functions operate from opposite physical assumptions—statistical geometry versus semi-flexible energetics—which is exactly the kind of complementarity that justifies running more than one method. ITD DOCK contributes a third perspective based on explicit AMBER electrostatics that neither of the other two engines evaluates directly, but its role in the consensus is asymmetric: as an in-house prototype without external benchmarking, its scores are used as a ranking signal rather than as a calibrated affinity estimator. The practical consequence is that the consensus rank is robust to occasional ITD DOCK failures (such as the three observed for ACS), since those complexes receive poor ITD DOCK ranks that are outweighed by consistent signals from the other two engines.

If any two of these were strongly correlated (r>0.8), that would mean they were measuring the same thing and one of them could be dropped without losing information. The near-zero correlations we observed confirm that all three are contributing something distinct to the final ranking, supporting the added computational cost. Low correlation between docking scoring functions is not a red flag; it is a well-documented property of consensus docking approaches, where combining methods that evaluate different physical dimensions of binding reduces shared systematic errors and improves true positive enrichment compared to any single method [37,38,39,40,41].

### 2.9. Why Sequence Features Do Not Work

Both the Random Forest (Analysis A, $R^{2}=-0.012$) and the Transformer ($R^{2}=0.002$) failed to predict docking scores from sequence features using the current computational framework. The convergence of two architecturally distinct modeling approaches on the same null result suggests that this reflects a property of the data rather than a limitation of any particular algorithm, though we cannot fully exclude that alternative feature representations or more expressive architectures might capture partial signal not detected here.

It is important to distinguish two different claims. The first is a biological claim: nucleotide sequence does determine three-dimensional structure, which in turn determines binding affinity. This is well established and not challenged by our results. The second is a computational claim specific to this study: the docking scores produced by HDOCK Lite and HADDOCK3, which are mathematical approximations derived from force-field energetics and knowledge-based potentials applied to pre-computed static conformations, do not appear to be predictable from sequence-derived features at the level of accuracy required for useful pre-screening. These are simplified models of a complex physical process, and the gap between sequence information and their numerical output is larger than current machine learning approaches can bridge without explicit three-dimensional structural evaluation. Whether higher-fidelity docking methods or more sophisticated sequence encodings might narrow this gap remains an open question.

The ΔG result makes this even clearer. Folding stability (r=0.012, p=0.23 with HADDOCK score) is independent of binding affinity, and it makes sense: an aptamer can fold stably into a shape that is completely wrong for the target, and a less stable aptamer can fold into exactly the right shape. Stability and complementarity are orthogonal properties. We use ΔG as a pre-filter to remove structurally unstable sequences before investing compute on docking, but it is not a proxy for binding.

Analysis B ($R^{2}=0.991$) was included as a positive control, not as a scientific finding: three of the four features used (VdW, Elec, Desolv) appear directly in the HADDOCK score formula with fixed coefficients, and BSA correlates strongly with the composite score even though it is not a formula term. The model’s near-perfect performance is, therefore, expected by construction. Its purpose is to verify that the Random Forest algorithm functions correctly when a signal is present, making the failure of Analysis A interpretable as a genuine absence of predictive content rather than a methodological artifact [42].

### 2.10. What the Dataset Is Good for

The 9813-complex dataset with three independent scoring dimensions per aptamer is the main output of this work. A realistic near-term application for machine learning in this context is not to replace docking but to use a sequence-based model as a coarse pre-filter: even a weakly predictive model for docking scores could reduce a library of $10^{5}$ candidates to $10^{4}$ before full tripartite evaluation, reducing total compute time by an order of magnitude. Building that kind of model requires labeled data that captures the multi-dimensional binding landscape, which is exactly what the combination of three orthogonal scoring functions provides. The addition of ACC synthase data for the same aptamer library adds a cross-target dimension that single-target datasets cannot replicate.

### 2.11. The Design Rules Are a Starting Point

The decision tree thresholds (Elec <−221 kcal/mol, BSA >1907 Å^2^, VdW <−87 kcal/mol for the top-scoring cluster) should be interpreted as empirical guidelines within this dataset, not universal laws of RNA-protein binding. They are derived from HADDOCK3 scores and will be specific to the scoring function, the target, and the aptamer library used here. Their practical value is in providing a rational basis for prioritizing candidates for experimental testing, not for designing aptamers from scratch.

### 2.12. Pilot ACC Synthase Validation and the Cross-Target Picture

The pilot ACS screening (n=97) suggests that the pipeline generalizes to a structurally distinct enzyme with a different active site, though full confirmation will require processing the complete library against ACS. The slight HDOCK Lite–HADDOCK3 correlation for ACS (ρ=0.235) that was absent for ACO is interesting: it may reflect that ACS has a more rigid active site where shape complementarity and induced-fit corrections happen to pull in the same direction. More data would be needed to say this with confidence.

The identification of aptamer_87930 and aptamer_82981 as preliminary dual-active candidates is the most actionable result of the pilot ACS screening. Both enzymes were screened with the same aptamer library, and the 97 valid ACS complexes were compared directly against the full ACO dataset, so the cross-target comparison is direct. The overlap between top-ten lists should be confirmed against the full ACS library before being treated as a robust finding, but these two aptamers represent a reasonable starting point for experimental prioritization if the research goal is simultaneous inhibition of both pathway steps.

### 2.13. Limitations and Future Directions

This work is a methodological proof-of-concept. The pipeline is validated computationally; none of the candidates have been synthesized or tested experimentally. All binding predictions are based on docking scores from computational models, and translating those into actual inhibitory activity requires experimental follow-up. The intended validation roadmap includes: (i) surface plasmon resonance (SPR) or isothermal titration calorimetry (ITC) binding assays to determine dissociation constants for the top-ranked aptamers against purified ACO and ACS; (ii) enzyme inhibition assays measuring ethylene production in cell-free systems in the presence of candidate aptamers; and (iii) for the two putative dual-active candidates (aptamer_87930 and aptamer_82981), parallel testing against both enzymes to confirm the predicted dual-inhibition profile. These experiments are planned as the immediate next phase of the research program but could not be completed within the time frame of this study.

ITD DOCK is an in-house prototype under active development and has not been validated against external benchmarks such as the CAPRI or HADDOCK community challenges. Its role in the current pipeline is to provide a third ranking dimension based on explicit AMBER electrostatics; its absolute numerical scores should not be interpreted as calibrated binding affinities. The three anomalous positive scores observed for ACC synthase (excluded from the ACS analysis) illustrate this limitation: the global FFT search occasionally fails to find a physically reasonable pose, particularly for targets with active site geometries that differ significantly from those in the ACO training set for which the engine was primarily tuned.

A significant limitation is that the full tripartite evaluation against ACC synthase could not be completed within the time frame of this study. Processing 14,659 aptamers against a second target through all three engines at 55 min per complex requires approximately 13,390 additional CPU-hours. This computation is currently ongoing in parallel on the Clúster Huapáctic at LNS-BUAP and on the local workstations at TecNM/Instituto Tecnológico de Durango, with an estimated completion timeline of four to six months from the date of this submission. The 97-aptamer pilot screening reported here is sufficient to demonstrate framework transferability and identify preliminary selectivity trends, but it is explicitly not a complete ACS dataset comparable in depth to the ACO analysis. Upon completion of the full-library ACS evaluation, the complete cross-target analysis will be reported in a follow-up study. Similarly, 4846 of the 14,659 ACO aptamers currently have only HADDOCK3 scores; completing ITD DOCK and HDOCK Lite for those complexes also represents several months of work that could not be finalized for this publication. Both are the immediate next computational priorities. The train/test split for all machine learning models was purely random (random_state=42) rather than controlled for sequence similarity; a similarity-aware partition would provide a more rigorous test of model generalization to structurally novel aptamers and is recommended for future work. Errors in RNAComposer tertiary structure predictions carry forward through all downstream steps, and rigid-body treatments in ITD DOCK and HDOCK Lite miss binding modes that depend on large conformational changes not captured by HADDOCK3’s semi-flexible refinement alone.

A realistic path from computational aptamer candidates to practical postharvest application requires addressing several biological challenges not covered by this pipeline. RNA aptamers are susceptible to nuclease degradation in plant tissues and external environments, which would require chemical modifications—such as 2′-fluoro or 2′-O-methyl substitutions on the ribose—to improve stability [12,13]. Delivery into plant cells or intercellular spaces raises additional challenges around cell wall penetration, off-target binding to non-enzyme RNA-binding proteins, and potential environmental persistence if used in field applications. Cost of synthesis and regulatory status for agricultural use of nucleic acid-based agents would also need to be assessed before any practical deployment. These considerations are beyond the scope of the current computational study but should inform the design of any downstream experimental program.

Future directions include: (1) completing the tripartite evaluation for the 4846 ACO aptamers currently at HADDOCK3-only status, yielding 14,659 fully annotated ACO complexes; (2) running the full pipeline against ACC synthase with the same 14,659 aptamers to enable direct cross-target comparison at scale; (3) molecular dynamics simulations for the top dual-inhibitor and enzyme-selective candidates; (4) experimental characterization through binding assays; (5) training sequence-based pre-screening models on the complete dual-target dataset; and (6) extension to other plant hormone biosynthesis targets.

## 3. Materials and Methods

The pipeline was applied primarily to ACC oxidase (PDB ID: 1W9Y). We ran all 9813 aptamer candidates through all 3 docking engines. For the cross-target comparison, a subset of 100 aptamers was docked against ACC synthase (PDB ID: 1YNU) using the exact same protocol; 3 complexes with anomalous positive ITD DOCK scores were subsequently excluded, leaving 97 valid ACS complexes.

### 3.1. Stochastic Sequence Generation and Thermodynamic Filtering

We wrote a Python 3.11 generator that produced $10^{6}$ unique RNA sequences with lengths between 20 and 80 nucleotides. Most random sequences fold poorly, so we filtered by thermodynamic stability using ViennaRNA [43,44] to compute the minimum free energy (ΔG) for each sequence. The 10,000 candidates with the lowest ΔG went on to three-dimensional modeling. After processing through RNAComposer and quality control, 9825 structures were successfully generated (175 failed due to RNAComposer server errors or invalid secondary structure notation). We then removed 12 complexes where the HADDOCK3 restraint energy was anomalously high (Restraints > 100 kcal/mol), consistent with docking failures rather than real binding, leaving 9813 valid complexes for all downstream analyses. The thermodynamic pre-filter is not meant to predict binding (we show later that ΔG has essentially zero correlation with docking score), but it prevents wasting computational resources on sequences that would fall apart structurally.

### 3.2. Automated Three-Dimensional Structural Modeling

Docking requires three-dimensional coordinates, not just sequences. We used RNAComposer [45], which has been benchmarked on CASP15 and remains a practical option for large-scale RNA modeling [46], to generate PDB structures from the dot-bracket secondary structures output by ViennaRNA.

All structures went through a cleaning step using PDB Tools. For the receptor (ACC oxidase, PDB 1W9Y), we retained only chain A and applied pdb_delhetatm to remove all HETATM records, including crystallographic water molecules, the catalytic iron ion, ascorbate, and the bound substrate ACC. A selenomethionine-to-methionine substitution (MSE→MET) was applied where present. The processed receptor, therefore, represents the apo enzyme in the absence of cofactors and substrate, which is standard practice for large-scale in silico aptamer screening pipelines [14,18,26]. The command pipeline was: pdb_delhetatm | pdb_chain -A | pdb_tidy | sed s/MSE/MET/g. The identical preparation protocol was applied to the ACC synthase receptor (PDB 1YNU, chain A retained), including removal of the pyridoxal phosphate (PLP) cofactor and crystallographic waters via pdb_delhetatm, yielding an apo form of ACS for the pilot cross-target screening. For aptamers, we extracted chain B and standardized the nucleotide names from RNA convention (RA→A, RG→G, RC→C, RU→U) with pdb_chain -B | pdb_delhetatm | pdb_tidy | sed. Hydrogen atoms and partial charges were added subsequently by each docking engine using its own force field: AMBER ff99bsc0 for ITD DOCK, knowledge-based potentials for HDOCK Lite, and CNS parameters for HADDOCK3. No explicit protonation state assignment was performed prior to docking; protonation was handled internally by each engine.

### 3.3. Engine 1: ITD DOCK (GPU-Accelerated Physics)

ITD DOCK is a docking engine we built in PyTorch 2.1, designed from the start to run on GPU hardware. It combines a global FFT search with AMBER-based local refinement. We ran all 9813 aptamers through it against ACC oxidase. The engine is under active development and should be considered a working prototype: it has not yet undergone the external benchmarking that HDOCK Lite and HADDOCK3 have received over years of community use. Its scores contribute a third independent ranking perspective based on explicit AMBER electrostatics, but numerical values from ITD DOCK alone should be interpreted with this caveat in mind. In particular, a small fraction of complexes produced anomalous positive scores for ACC synthase (3 out of 100), which we attribute to failures in the global FFT search rather than real binding predictions; these were excluded from the ACS analysis.

#### 3.3.1. Multi-Channel Gaussian Voxelization

Atoms are projected onto a 4-channel volumetric grid $G\in R^{4\times 96\times 96\times 96}$ with 0.8 Å spacing. The four channels encode: (1) Van der Waals density ($r_{vdw}^{3}$); (2) electrostatic partial charges *q*, derived from AMBER ff99bsc0 for RNA nucleotides and from AMBER ff99SB for protein residues [47]; (3) a hydrophobicity mask for carbon atoms (|q|<0.1); and (4) Lennard-Jones depth parameters ϵ. Atoms are spread onto the grid using a Gaussian kernel ($G\left(r\right)=\exp(-||r-r_{atom}|{|}^{2}/2\sigma^{2})$) to avoid sharp discontinuities.

#### 3.3.2. FFT Correlation and Global Search

Interaction scores are computed by convolution in the frequency domain:

(1) $$\left(R★L\right)\left(x\right)=F^{-1}\left\{F\left(R\right)\cdot \bar{F(L)}\right\}$$

where ★ denotes convolution and the overline is the complex conjugate. Grids are padded to $192^{3}$ to prevent wrap-around artifacts. We sample rotational space at $15^{\circ }$ intervals (approximately 12,000 orientations) using CUDA-accelerated batch FFT to find the global energy minimum efficiently.

#### 3.3.3. Gradient-Based Refinement with AMBER Force Field

The 50 best-scoring poses from the FFT stage are then refined locally for 150 gradient descent steps. We use a KDTree to find neighbor pairs within $r_{cutoff}=6.0$ Å and evaluate:

(2) $$E_{interaction}=E_{vdW}+E_{elec}+E_{hbond}$$

(3) $$\begin{array}{cc} E_{vdW} & =\sum_{i,j}\epsilon_{ij}\left[{\left(\frac{R_{min,ij}}{r_{ij}}\right)}^{12}-2{\left(\frac{R_{min,ij}}{r_{ij}}\right)}^{6}\right] \end{array}$$

(4) $$\begin{array}{cc} E_{elec} & =\sum_{i,j}\frac{q_{i}q_{j}}{4\pi \epsilon_{0}\epsilon_{r}r_{ij}} \end{array}$$

A soft-core repulsion term is applied when $E_{vdW}>10$ kcal/mol to prevent atoms from overlapping during minimization.

### 3.4. Engine 2: HDOCK Lite (Geometric Consensus)

HDOCK Lite [48] is a standalone version of the HDOCK server, trimmed down for local deployment. We ran all 9813 aptamers through it. The reason it is useful here is that its scoring function, ITScore-PR [49,50], comes from a completely different place than AMBER: instead of computing physical forces, it estimates interaction likelihood from statistical patterns in known protein-RNA crystal structures. If a contact geometry appears often in the PDB, it gets a favorable score regardless of whether the physics engine would flag it as particularly strong.

HDOCK Lite uses a hierarchical FFT search [51,52] focused on shape complementarity, followed by ITScore-PR scoring:

(5) $$E_{HDOCK}=\sum_{i,j}u_{ij}\left(r\right)$$

where $u_{ij}\left(r\right)$ are the pair potentials between atom types *i* and *j* at distance *r*. The result is a score that captures what “looks like a real binding pose” based on structural precedent, not electrostatics.

### 3.5. Engine 3: HADDOCK3 (Semi-Flexible Refinement)

HADDOCK3 [53] is the most computationally expensive of the three but also the most physically realistic: it lets the RNA backbone and protein side chains move during refinement using CNS as its backend. All 9813 candidates were processed through it.

The docking in HADDOCK3 was not global but directed to the enzyme active site using CNS distance restraints. For ACC oxidase, restraints were defined on the iron-coordinating facial triad residues His177, Asp179, and His234 (chain A), which constitute the catalytic core of the non-heme Fe(II) active site. For ACC synthase, restraints targeted Asn202, Tyr233, Lys273, and Arg407 (chain A), with Lys273 being the residue that forms the Schiff base with the PLP cofactor. These residues were selected based on published crystallographic and mechanistic studies of each enzyme [19,20]. The restraint format was: assign (resi X and segid A)(segid B) 2.0 2.0 0.0, enforcing proximity between the aptamer and the specified active-site residues while allowing HADDOCK3 to optimize the exact binding geometry. Note that while the catalytic Fe(II) ion and PLP cofactor were removed from the receptor structures prior to docking (as described above), the restraints target the protein residues that coordinate these cofactors, thereby preserving the geometric definition of the active site. This active-site-directed approach contrasts with ITD DOCK and HDOCK Lite, which perform unrestricted global FFT searches across the entire protein surface. The protocol then progressively introduces flexibility: it0 performs rigid-body energy minimization within the restrained binding region, it1 applies semi-flexible simulated annealing in torsion angle space where protein side chains and the RNA backbone at the binding interface are allowed to move, and itw carries out final refinement in explicit TIP3P water. HADDOCK3 is, therefore, the only engine in the pipeline that is both active-site-directed and capable of capturing induced-fit effects. The scoring function is:

(6) $$S_{haddock}=1.0E_{vdw}+0.2E_{elec}+1.0E_{desol}+0.1E_{air}$$

The desolvation term ($E_{desol}$) matters especially for RNA, since displacing water from the phosphate backbone carries a thermodynamic cost that rigid methods ignore. HADDOCK3 also outputs the individual energy components ($E_{vdw}$, $E_{elec}$, $E_{desol}$, BSA), which is what makes the feature importance analysis possible.

### 3.6. AptaDock: Integrated Pipeline Software

Running this pipeline manually across thousands of aptamers is not realistic, so we developed AptaDock [54], a desktop application (Python 3.11, tkinter) that automates all 13 steps through a graphical interface. The first module handles aptamer processing: sequence generation, thermodynamic filtering, G-quadruplex scoring, batch submission to RNAComposer via Selenium, structure download, and PDB cleaning. The second module runs the three docking engines.

The interface shows each step as a card that can be locked, active, or completed. If a run is interrupted, the application resumes from where it left off without reprocessing completed aptamers. HDOCK Lite and HADDOCK3 require Linux because their underlying binaries (Fortran and CNS) are only available there; ITD DOCK runs on both Windows and Linux. AptaDock is available on request from the corresponding author for academic use.

### 3.7. Computational Infrastructure and Parallelization

We ran the pipeline on three identical workstations (AMD Ryzen 7 7000 Series, 16 GB DDR5, NVIDIA RTX 4070) working in parallel, which cut total wall-clock time by a factor of three. Approximate runtimes per aptamer: ITD DOCK ≈ 2 min, HDOCK Lite ≈ 8 min, HADDOCK3 ≈ 45 min, totaling around 55 min per complex for the full tripartite evaluation.

Additional HADDOCK3 simulations were carried out on the Clúster Huapáctic at the Laboratorio Nacional de Supercómputo del Sureste de México (LNS-BUAP) [55], which comprises 84 thin compute nodes (2 × 12-core CPU, 128 GB RAM each) and 2 fat nodes (2 × 12-core CPU, 256 GB RAM each), running Rocky Linux 8.7. We processed 4846 additional aptamer complexes through HADDOCK3 on these nodes. The current tripartite dataset comprises the 9813 aptamers for which all three engine scores are available; the remaining 4846 HADDOCK3-only evaluations are being completed to form a unified set of 14,659 fully annotated complexes.

### 3.8. Consensus Metric and Ranking

To combine the three scores into a single ranking, we used normalized rank aggregation. For each aptamer *i* and engine *X*:

(7) $$r_{X}\left(i\right)=\frac{R_{X}\left(i\right)}{N}$$

where $R_{X}\left(i\right)$ is the rank in engine *X* (1 = best) and *N* is the total number of candidates. The consensus score is the average across engines:

(8) $$C\left(i\right)=\frac{1}{3}\left(r_{ITD}\left(i\right)+r_{HDOCK}\left(i\right)+r_{HADDOCK}\left(i\right)\right)$$

Lower consensus scores mean better candidates. Ranking rather than averaging raw scores avoids the problem of comparing values on completely different scales.

### 3.9. Transformer-Based Sequence-to-Score Model

To test whether a deep learning model could learn to predict docking scores from sequence features, we trained a multi-task Transformer to predict both HADDOCK score and HDOCK score simultaneously. The architecture uses 6 encoder layers with $d_{model}=256$, 8 attention heads, feed-forward dimension 1024, Rotary Position Embeddings (RoPE), SwiGLU activations, and RMSNorm normalization. Dropout was 0.2.

The input combines 3-mer tokenization (vocabulary: 4096 buckets) with 40 numerical features from sequence and secondary structure: nucleotide frequencies, dinucleotide content, GC content, folding energy (ΔG), net charge, and secondary structure descriptors. No energy components from HADDOCK3 or HDOCK Lite were included, since those are only available after docking and the point of the model would be to avoid running docking in the first place. Training ran for up to 30,000 steps (batch 32, learning rate $10^{-4}$, linear warmup, weight decay 0.01, AMP), with a multi-task loss weighting HADDOCK score at 3.0 and HDOCK score at 1.0. An 80/20 train/test split was used with early stopping (patience = 15 epochs).

### 3.10. Statistical Analysis and Feature Importance

We ran two complementary Random Forest analyses [35] on the full ACO dataset (n=9813), both predicting HADDOCK score but from different feature sets.

*Analysis A: Sequence-Only Baseline.* This model used 48 features derivable purely from sequence and secondary structure: nucleotide and dinucleotide frequencies, GC content, thermodynamic folding energy (ΔG), net charge, and secondary structure descriptors (paired fraction, hairpin count, stem count, structural complexity). HADDOCK3 energy components were excluded by design. This is the non-circular test: can you predict docking scores without any three-dimensional information about the complex?

*Analysis B: Positive Control.* As a positive control to verify that the Random Forest algorithm can capture mathematical relationships when a real signal is present, we trained a second model using only the four HADDOCK3 energy outputs (VdW, Elec, Desolv, BSA) as features. Three of these four terms (VdW, Elec, Desolv) appear directly in the HADDOCK score formula with fixed coefficients; BSA is not a formula component but correlates strongly with the composite score because larger buried interfaces systematically drive more favorable interaction energies. As a result, this model is expected to perform well by construction: it is not discovering new biology but recovering a known mathematical structure. Its role here is purely contrastive: by comparing Analysis A (sequence features, no three-dimensional information) against Analysis B (HADDOCK3 energy outputs, full three-dimensional information), we can quantify how much predictive content is only accessible after docking.

Both models used 500 estimators and an 80/20 train/test split. All machine learning models were implemented using scikit-learn 1.3. We also computed Pearson and Spearman correlations [56] for each feature individually, applied Principal Component Analysis (PCA) [57] to characterize the dataset structure, and ran K-means clustering to identify subgroups with distinct binding profiles. The number of clusters was selected by evaluating three complementary metrics across k=2 to k=10: the elbow method, the silhouette score, and the Davies–Bouldin index (Figure 12). k=5 achieved the global minimum of the Davies–Bouldin index (DB=2.720, versus 2.871 for k=3 and 2.847 for k=6), a local maximum in the silhouette score (s=0.068, versus 0.066 for k=4 and 0.059 for k=6), and corresponds to the inflection region of the elbow curve. Cluster stability was confirmed with 10 independent random initializations (n_init=10).

## 4. Conclusions

We built a pipeline that screens large aptamer libraries against ACC oxidase and ACC synthase using three largely complementary docking engines: ITD DOCK (GPU-accelerated AMBER physics), HDOCK Lite (knowledge-based geometric scoring), and HADDOCK3 (semi-flexible refinement with energy decomposition). Applied to ACC oxidase with 9813 aptamers, we confirmed that the three engines produce largely complementary, weakly correlated scores, which supports their combined use as a consensus rather than averaging redundant information.

We then asked whether it would be possible to skip docking and predict docking scores from sequence features. A Random Forest trained on 48 sequence-derived features (Analysis A) achieved $R^{2}=-0.012$ (RMSE = 21.79 kcal/mol); it could not even beat the trivial prediction of the dataset mean. A Transformer trained on the same class of features confirmed the result ($R^{2}=0.002$, collapse to the mean). The convergence of two independent model families on the same null result rules out architecture-specific failure and confirms that sequence information is simply not sufficient for docking score prediction in this system. For contrast, a Random Forest trained on the HADDOCK3 energy components (Analysis B, positive control) achieved $R^{2}=0.991$, confirming the algorithm works when the signal is present. The information simply does not exist until the docking is run.

The univariate analysis tells the same story more directly: BSA (r=−0.749), VdW (r=0.703), and Elec (r=0.690) are strong individual predictors of the HADDOCK score, while ΔG (r=0.012, p=0.23) and all sequence features are essentially noise. PCA showed that 10 principal components are needed to explain 65.7% of dataset variance, and K-means clustering identified five aptamer groups with distinct binding profiles. Decision tree analysis suggests that the most promising candidates combine strong electrostatics (Elec <−221 kcal/mol), a large buried interface (BSA >1907 Å^2^), and favorable VdW contacts (VdW <−87 kcal/mol) simultaneously.

A pilot cross-target screening on ACC synthase (n=97 valid complexes, after excluding three ITD DOCK failures) showed that the engine independence pattern holds for a second target and identified two putative dual-active candidates, aptamer_87930 and aptamer_82981, that ranked in the top 10 for both enzymes and represent the highest-priority targets for experimental follow-up once the full ACS library has been evaluated. 80% of the top-ranked positions were enzyme-selective. The full 14,659-aptamer evaluation against ACC synthase is ongoing and was not completed within the time frame of this study.

The entire workflow runs through AptaDock [54], a desktop application that automates all 13 steps from sequence generation to consensus ranking, with automatic resumption from interruptions and built-in dependency handling. The dataset generated here provides the foundation for developing pre-screening models that could reduce the computational load of future large-scale aptamer campaigns.

## Figures and Tables

**Table 1:** Correlation analysis between docking engines for ACC oxidase—pilot sample (n=100, randomly sampled).

Engine Pair	Pearson *r*	Spearman ρ	Kendall τ
ITD DOCK vs. HDOCK Lite	0.012 (*p* = 0.907)	−0.003 (*p* = 0.981)	0.001 (*p* = 0.986)
ITD DOCK vs. HADDOCK3	−0.026 (*p* = 0.800)	−0.046 (*p* = 0.652)	−0.027 (*p* = 0.688)
HDOCK Lite vs. HADDOCK3	−0.165 (*p* = 0.102)	−0.196 (*p* = 0.051)	−0.139 (*p* = 0.040) *

* Statistically significant at α=0.05 level. All other correlations are non-significant. The borderline HDOCK Lite–HADDOCK3 association was re-evaluated on the full dataset (Table 2); it did not replicate, indicating a sampling artefact in the pilot.

**Table 2:** Correlation analysis between docking engines for ACC oxidase—full dataset (n=9813).

Engine Pair	Pearson *r*	Spearman ρ	Kendall τ
ITD DOCK vs. HDOCK Lite	−0.007 (*p* = 0.480)	−0.007 (*p* = 0.468)	−0.005 (*p* = 0.474)
ITD DOCK vs. HADDOCK3	0.009 (*p* = 0.385)	0.003 (*p* = 0.783)	0.002 (*p* = 0.780)
HDOCK Lite vs. HADDOCK3	−0.008 (*p* = 0.426)	−0.013 (*p* = 0.210)	−0.009 (*p* = 0.206)

All correlations are non-significant (p>0.05 for all three metrics). The borderline correlation observed in the pilot sample (HDOCK Lite–HADDOCK3, Kendall τ=−0.139, p=0.040) did not replicate at full scale (τ=−0.009, p=0.206), confirming it was a small-sample artefact. These results provide strong statistical confirmation that the three engines contribute largely complementary, non-redundant information.

**Table 3:** Top features by random forest importance (ACC oxidase, n=9813): energy-component confirmation model.

Rank	Feature	Importance
1	Elec	0.463
2	VdW	0.268
3	BSA	0.175
4	Desolv	0.094
5–48	Composition features *	<0.001 each
	(dinucleotides, GC content,	
	structure complexity, etc.)	

* Results from the energy-component confirmation model (Analysis B). The sequence-only baseline model (Analysis A, 48 features) achieves $R^{2}=-0.012$ (RMSE = 21.79 kcal/mol), indicating performance below the trivial mean predictor. Analysis B achieves $R^{2}=0.991$. The gap in predictive capacity quantifies the information accessible only through docking.

**Table 4:** Transformer validation metrics on held-out test set (20%, n=1963).

Target	R^2^	Pearson *r*	MAE	RMSE	Range
HADDOCK score	0.002	0.048 (*p* = 0.073)	16.9 kcal/mol	23.0 kcal/mol	267.5 kcal/mol
HDOCK score	0.014	0.118 (*p* < 0.001)	25.9	33.1	249.5

HADDOCK score = composite semi-flexible refinement score (kcal/mol); HDOCK score = knowledge-based statistical potential score (dimensionless arbitrary units; not a physical energy). MAE, RMSE, and Range for HADDOCK score are in kcal/mol; values for HDOCK score are in arbitrary units.

**Table 5:** Correlation analysis between docking engines for ACC synthase (n=97, after excluding three ITD DOCK failures).

Engine Pair	Pearson *r*	Spearman ρ	Kendall τ
ITD DOCK vs. HDOCK Lite	0.077 (*p* = 0.453)	0.107 (*p* = 0.295)	0.067 (*p* = 0.331)
ITD DOCK vs. HADDOCK3	0.043 (*p* = 0.672)	−0.070 (*p* = 0.496)	−0.052 (*p* = 0.447)
HDOCK Lite vs. HADDOCK3	0.178 (*p* = 0.081)	0.235 (*p* = 0.021) *	0.170 (*p* = 0.014) *

* Statistically significant at α=0.05 level. All other correlations are non-significant.

**Table 6:** Cross-target selectivity: top-ten consensus candidates.

Category	Count	Aptamer IDs
Putative Dual-Active (both ACO and ACS)	2	aptamer_87930, aptamer_82981
ACO-Selective	8	aptamer_50836, aptamer_55059, aptamer_4057, aptamer_29394, aptamer_37966, aptamer_83927, aptamer_78309, aptamer_63141
ACS-Selective	8	aptamer_19833, aptamer_68030, aptamer_14856, aptamer_44856, aptamer_53173, aptamer_24617, aptamer_10119, aptamer_52632

## Data Availability

The docking datasets presented in this study are available on request from the corresponding author due to ongoing research activities. The AptaDock source code is hosted in a private GitHub repository and is available upon reasonable request to the corresponding author for academic purposes. Executable files, user documentation, and representative datasets are provided alongside repository access.

## Abbreviations

ACO	ACC oxidase
ACS	ACC synthase
ACC	1-aminocyclopropane-1-carboxylic acid
RNA	Ribonucleic acid
DNA	Deoxyribonucleic acid
SELEX	Systematic Evolution of Ligands by Exponential Enrichment
FFT	Fast Fourier Transform
GPU	Graphics Processing Unit
CUDA	Compute Unified Device Architecture
MFE	Minimum Free Energy
PDB	Protein Data Bank
AMBER	Assisted Model Building with Energy Refinement
CNS	Crystallography & NMR System
SPR	Surface Plasmon Resonance
ITC	Isothermal Titration Calorimetry
SAM	S-adenosyl-L-methionine
1-MCP	1-methylcyclopropene
PCA	Principal Component Analysis
BSA	Buried Surface Area
